# Pharmacobiological Approach for the Clinical Development of Ruxolitinib in Myeloproliferative Neoplasms

**DOI:** 10.4274/tjh.2013.0265

**Published:** 2015-05-08

**Authors:** Eylem Eliaçık, Ayşe Işık, Salih Aksu, Ayşegül Üner, Yahya Büyükaşık, Nilgün Sayınalp, Hakan Göker, Osman İ. Özcebe, İbrahim C. Haznedaroğlu

**Affiliations:** 1 Hacettepe University Faculty of Medicine, Department of Hematology, Ankara, Turkey; 2 Hacettepe University Faculty of Medicine, Department of Pathology, Ankara, Turkey

**Keywords:** Myeloproliferative neoplasms, Ruxolitinib, Myelofibrosis

## Abstract

Ruxolitinib, a JAK1 and JAK2 inhibitor drug, has recently been approved for the treatment of patients with high- or intermediate-risk myelofibrosis with symptomatic splenomegaly. Ruxolitinib is the first clinically useful targeted therapy in Philadelphia chromosome-negative myeloproliferative neoplasms (MPNs). The aim of this paper is to indicate pharmacobiological aspects of ruxolitinib within the potential context of MPNs. Pharmacobiological assessments, in addition to knowledge of the risk profile for ruxolitinib in MPNs, are required. We propose hypotheses based on our experience in a splenectomized MPN patient with hyperproliferative bone marrow and moderate fibrosis receiving ruxolitinib. We believe that a true clinical development approach for this drug should include pharmacobiological assessments for ruxolitinib in addition to the disease risk profile of MPNs.

## Abstract (tr)

Ruxolitinib, JAK1 ve JAK2 inhibitörü olarak işlev gören bir ilaçtır. Semptomatik splenomegalisi olan orta- veya yüksek-risk myelofibrozis hastalarında kullanımı uluslararası onam almıştır. Bu bağlamda ruxolitinib, Philadelphia kromozomu negatif myeloproliferatif neoplaziler (MPN) için klinik yararı gösterilen ilk hedefe yönelik ajan konumundadır. Bu yazının amacı, ruxolitinibin MPN’nin klinik tablolarındaki potansiyel kullanım alanları konusunda farmakobiyolojik yönleri tartışmaktır. Ruxolitinib onamları başlıca hastalık risk faktörleri üzerinden yapılmaktadır. Ancak klinik kullanımda hastalığın ve ilacın farmakobiyolojik yönlerini de dikkate alma gerekliliği vardır. Bu hipotezimizi tartışırken splenektomize bir MPN hastamızda, hiperproliferatif bir kemik iliği ve orta derecede fibrozis mevcutken uyguladığımız ruxolitinib tedavisinden elde ettiğimiz deneyimlere dayandık. İlacın gelecekte klinik geliştirilmesi gerçekleştirilirken MPN risk profili yanı sıra farmakobiyolojik değerlendirmelerin de yapılması gerektiği düşüncesindeyiz.

## INTRODUCTION

Ruxolitinib, a JAK1 and JAK2 inhibitor drug, has recently been approved for the treatment of patients with high- or intermediate-risk myelofibrosis (MF) with symptomatic splenomegaly [1]. This approval in MF depends upon 2 different phase 3 randomized clinical trials (RCTs), namely COMFORT-I and COMFORT-II. COMFORT-I compared ruxolitinib with a placebo in 309 patients with MF, whereas COMFORT-II compared the drug with the best-available therapy (mostly hydroxyurea) in 219 MF patients. Both of the RCTs attained the primary endpoint of >35% reduction in spleen size, as measured by imaging techniques, at 24 or 48 weeks after ruxolitinib treatment initiations [2,3]. Clinical development of ruxolitinib is currently focused on the Philadelphia-negative myeloproliferative neoplastic disorders (Ph -MPNs) [4].

Ruxolitinib is a “JAK-STAT signaling pathway inhibitor” targeted drug with predictable pharmacobiological actions. The main function of the JAK-STAT signaling pathway is cellular proliferation in health and disease. Ruxolitinib should thus be considered as an “anti-proliferative” medicine [4,5,6,7]. Ruxolitinib has the potential to inhibit neoplastic cellular proliferation of MPNs and can cause cytopenias due to its “anti-proliferative” effects in any hematopoietic lineage. The current view of ruxolitinib in MPNs is dependent upon mainly the disease risk profile of the given MPN entity. However, this risk-only approach is not sufficient and can cause the mechanistic wrong decision that ruxolitinib is unnecessary in low-risk MPN. Likewise, ruxolitinib may be considered as ineffective, useless, harmful, or dangerous in (very) high-risk advanced/terminal MPN due to cytopenias of the drug itself. Ruxolitinib could precipitate anemia, leukopenia, and thrombocytopenia in an already pancytopenic patient with MPN. However, there are some initial clues that ruxolitinib can reverse bone marrow fibrosis in MPN if the patient population (such as cases of hyperproliferative bone marrow with splenomegaly and peripheral cytosis) is carefully selected and long-term exposure to the drug (such as 48 months) is possible [8].

The aim of this paper is to indicate pharmacobiological aspects of ruxolitinib within the potential context of MPNs. Pharmacobiological assessments, in addition to clarification of the risk profile [9] for ruxolitinib in MPNs, are required. Current clinical challenges for ruxolitinib in MPNs are summarized in Table 1. Pharmacobiological assessments and risk profiles for ruxolitinib in MPNs are depicted in Table 2.

### Case Report, Methods, and Results: A Typical Myeloproliferative Neoplasms Case to Support the Hypothesis

**Figure 1:**
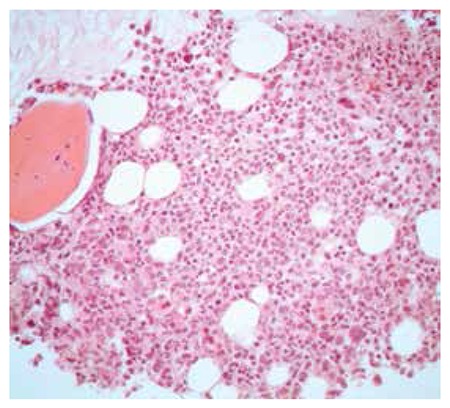
Hyperproliferative bone marrow of the patient diagnosed with JAK2V617F-positive polycythemia vera. Hypercellular bone marrow with grade 1 fibrosis and trilineage hyperplasia (100x).

**Figure 2:**
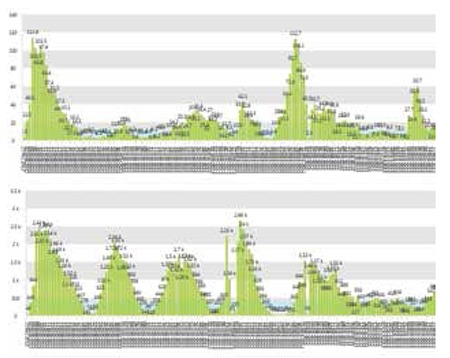
Peripheral white blood cell (upper panel) and Plt (lower panel) counts of the patient diagnosed with JAK2V617F-positive polycythemia vera. Control of the neoplastic cellular proliferation was obtained via a PEG-intron + ruxolitinib combination.

A 64-year-old female patient with elevated blood counts was evaluated in our hematology unit. Medical history revealed systemic hypertension for 35 years and the diagnosis of polycythemia vera (PV) 20 years earlier. In 1994, the patient underwent total gastrectomy and splenectomy in order to cure gastric cancer. The JAK2V617F mutation was also detected in due course. The patient was treated by phlebotomy only until 2003, and then hydroxyurea plus phlebotomy until 2008 to control the disease. At that time, the patient had acute respiratory failure due to hyperviscosity (Plt count over 4 million per mm3 and white blood cell (WBC) count of about 50,000 per mm3), deep vein thrombosis, gastrointestinal bleeding, nasal bleeding, and hydroxyurea-induced skin lesions. After emergency treatment with leukapheresis and Ara-C infusions, an effort was made to control the patient’s thrombocytosis with aa combination of hydroxyurea plus anagrelide. In the following years, the patient had several severe attacks due to hyperleukocytosis (WBC count reaching 120,000 per mm3) and extreme thrombocytosis (Plt count reaching 2 million per mm3) with hyperproliferative bone marrow (Figure 1) requiring intermediate doses of Ara-C infusions for 3-5 days. In June 2012, PEG-IFN treatment (180 µg/week) was initiated to control the PV. In October 2012, ruxolitinib (10 mg b.i.d.) was added to the treatment schedule and the dose of PEG-IFN was set as 90 µg in this dual combination. Complete blood counts were stable and the ongoing hemostatic systemic complications due to cytosis were successfully controlled with this combination treatment (Figure 2). 

Our patient represents a model of the ideal MPN population in which ruxolitinib should be administered, with hyperproliferative bone marrow with or without fibrosis and peripheral cytosis and organomegaly. Informed consent was obtained.

## DISCUSSION

Patients who have hyperproliferative bone marrow in any lineage (enhanced granulopoiesis, erythropoiesis, thrombopoiesis) plus/minus fibrosis with or without peripheral cytosis (hyperleukocytosis, polycythemia, thrombocytosis), and splenomegaly plus peripheral cytosis (hyperleukocytosis, polycythemia, thrombocytosis); all of the ruxolitinib arm of the RESPONSE trial patients; all ruxolitinib-receiving prefibrotic primary myelofibrosis (PMF, WHO 2008) patients; all ruxolitinib-receiving patients splenectomized for any reason; and any MPN patient to whom ruxolitinib was already administered within other trials (COMFORT-I, COMFORT-II, and others) on compassionate use could be registered and independently evaluated in the context of a ‘Ruxolitinib Pan-MPN Trial’. In this specific MPN patient population (Ruxolitinib Pan-MPN Registry), some critical clinical/laboratory evaluations including effects of ruxolitinib on white blood cell counts (control versus leukopenia) and leukostasis/infections, on hematocrit levels (control versus anemia) and hyperviscosity, on platelet levels (control versus thrombocytopenia) and leukostasis/thrombosis/hemorrhage, on hepatic enlargement and complications of portal hypertension (particularly after splenectomy), and on hyperproliferative bone marrow neoplastic cellular proliferation and fibrosis would be assessed. MPN disease risk categories of this specific MPN patient population should be detected, as well as the established clinically important ruxolitinib effects (reduction in spleen size and MPN symptoms). 

A Ruxolitinib Pan-MPN Phase II study should be performed if the Step 1 Ruxolitinib Pan-MPN Registry reveals that ruxolitinib can control hyperproliferative bone marrow (enhanced granulopoiesis, erythropoiesis, thrombopoiesis), fibrosis and/or peripheral cytosis (hyperleukocytosis, polycythemia, thrombocytosis), cytosis-related acute/sub-acute complications (hyperleukocytosis, polycythemia, thrombocytosis), or hepatic enlargement and complications of portal hypertension (particularly after splenectomy), or can decrease spleen size in PV and normalize bone marrow architecture in MPNs in the long term in a study population whose main inclusion criteria are any MPN patient with hyperproliferative bone marrow in any lineage AND bone marrow fibrosis AND splenomegaly AND peripheral cytosis in at least one lineage (hyperleukocytosis, polycythemia, thrombocytosis), and all of the MPN patients with PMF (WHO 2008). These proposals will be tested as hypotheses on efficacy. Hypotheses on safety, including that the degree of ruxolitinib-induced anemia, leukopenia, thrombocytopenia, and related complications is lower in hyperproliferative MPN and that the tolerability and adherence of ruxolitinib with proper dosage and duration is enhanced in patients with hyperproliferative MPNs, will also be tested. 

The development of any drug from bench side to clinic is very difficult and expensive. Therefore, proper scientific strategy is absolutely necessary during the design of clinical studies. Ruxolitinib is the first clinically useful targeted therapy in Ph -MPNs. We think that a true clinical development approach for this drug should include pharmacobiological assessments for ruxolitinib in addition to the disease risk profile of MPNs.

## Figures and Tables

**Table 1:**
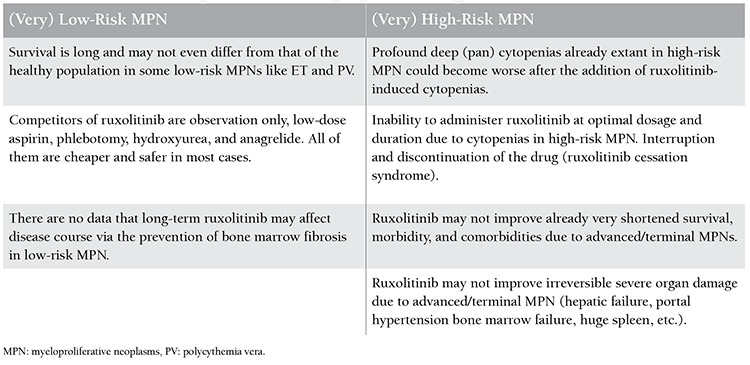
Current clinical challenges for ruxolitinib in myeloproliferative neoplasms (MPNs).

**Table 2:**
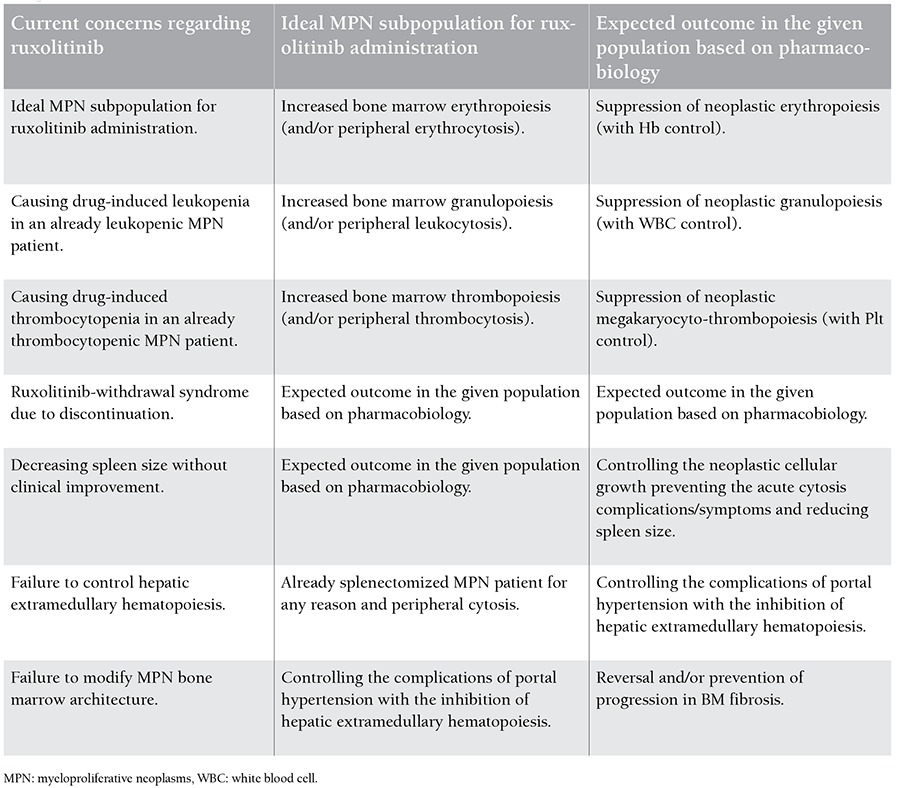
The need for pharmacobiological assessments in addition to the risk profile for ruxolitinib in myeloproliferative neoplasms (MPNs).
